# EZH2 knockdown suppresses the growth and invasion of human inflammatory breast cancer cells

**DOI:** 10.1186/1756-9966-32-70

**Published:** 2013-09-27

**Authors:** Zhaomei Mu, Hua Li, Sandra V Fernandez, Katherine R Alpaugh, Rugang Zhang, Massimo Cristofanilli

**Affiliations:** 1 Department of Medical Oncology, Thomas Jefferson University; and Kimmel Cancer Center, Philadelphia, PA 19107, USA; 2 Department of Medical Oncology, Fox Chase Cancer Center, Philadelphia, PA 19111, USA; 3 Wistar Institute, Philadelphia, PA 19104, USA

**Keywords:** Inflammatory breast cancer, EZH2, Cancer stem cell, Tumor spheroid formation

## Abstract

**Introduction:**

Inflammatory breast cancer (IBC) is the most metastatic variant of breast cancer with the poorest survival in all types of breast cancer patients and presently therapeutic targets for IBC are very limited. Enhancer of zeste homolog 2 (EZH2) is frequently expressed in human IBC and its expression positively correlates with worse clinical outcome. However, the molecular basis for EZH2 promoting IBC has not been explored. Here, we investigated the functional role of EZH2 in IBC cells by examining the effects of its knockdown on the formation of tumor spheroids and invasion of these cells *in vitro* and *in vivo* in an orthotopic xenograft model.

**Methods:**

SUM149 and a new IBC cell line-FC-IBC-02 derived from pleural effusion fluid of an IBC patient were used in this study. Specific knockdown of EZH2 was performed using short hairpin RNA (shRNA) specific to the human EZH2 gene. Cell growth and the formation of tumor spheroids were examined *in vitro*. The effects of EZH2 knockdown on IBC cell migration and invasion were examined by a Boyden chamber assay. For the *in vivo* tumor growth studies, IBC cells were orthotopically transplanted into the mammary fat pads of immunodeficient mice.

**Results:**

The results showed that EZH2 is expressed at higher levels in human IBC cell lines compared with normal human mammary epithelial cells, and the knockdown of EZH2 expression significantly suppressed cell growth and tumor spheroid formation of human IBC cells *in vitro.* In addition, EZH2 knockdown inhibited the migration and invasion of IBC cells. Significantly, EZH2 knockdown suppressed the angiogenesis and tumor growth of IBC cells *in vivo*.

**Conclusions:**

Our results provide direct evidence that EZH2 is critical for the formation of tumor spheroids and invasion of human IBC cells and could be a potential target for developing novel therapeutic strategies for human IBC.

## Introduction

Inflammatory breast cancer (IBC) is the most aggressive variant of locally advanced breast cancer, and currently accounts for 1% to 6% of all breast cancer patients in the United States [1,2]. IBC has shown the capacity to spread early, primarily through lymphatic channels and secondarily through blood vessels causing the specific inflammatory signs, such as diffuse erythema, edema, tenderness, and warmth [3,4]. IBC is difficult to detect as it does not present as a lump but rather occurs as tumor emboli. These IBC tumor emboli are non-adherent cell clusters that rapidly spread by a continuous passive dissemination, thus favoring both distal metastasis and local recurrence [5]. Primary treatment of patients with IBC is typically multimodal involving neoadjuvant combination chemotherapy followed by surgery, adjuvant chemotherapy, or radiotherapy [6]. Epidermal growth factor receptor (EGFR) and HER2 receptor overexpression have been demonstrated as prognosis and predictive factors in IBC [7,8]. Anti-HER2 therapies have shown benefit in IBC patients with HER2 amplification, which accounts for approximately 40% of IBC [9]. However, therapeutic options for patients with estrogen-receptor negative (ER-) and HER2 non-amplified IBC patients are very limited. Notably, IBCs are predominantly basal-like or triple-negative as demonstrated by the ER-negative, progesterone receptor (PgR)-negative and HER2 non-amplified status [10]. Thus, there is an urgent need to identify a new therapeutic strategy for IBC, particularly for triple-negative IBC.

Enhancer of zeste homolog 2 (EZH2) is the catalytic subunit of polycomb repressive complex 2 (PRC2), while noncatalytic subunits suppressor of zeste 12 (SUZ12) and embryonic ectoderm development (EED) are also necessary for its optimal activity [11]. EZH2 is upregulated in several types of cancers and has been implicated in regulating multiple cellular processes such as proliferation, differentiation, cell cycle, apoptosis, invasion, and self-renewal [12,13]. For example, EZH2 is often expressed at higher levels in breast cancer and its overexpression correlates with aggressiveness and poor prognosis [14-20]. We have recently shown that EZH2 is often expressed in IBCs and is a marker for poor prognosis in these patients [21]. Interestingly, there is a significant positive correlation between EZH2 expression and triple-negative status [21]. Strikingly, all triple-negative tumors were found positive for EZH2 expression. This expression pattern raises the possibility that EZH2 could represent a potential therapeutic target in IBC and, in particular, in triple-negative IBC that currently has very limited treatment options.

The clinical symptoms and biological features of IBC are very distinct from other types of breast cancer. Currently there are few human IBC cell lines available for studying this complex disease. We have recently developed a new IBC cell line FC-IBC-02 that was derived from the pleural effusion fluid of a woman with metastatic secondary IBC [22]. FC-IBC-02 cells are triple-negative, and form tumor spheroids in suspension culture, a characteristic of cancer stem cells, and recapitulate the tumor emboli, a signature sign of IBC in humans *in vivo* xenograft models. FC-IBC-02 cells also expressed some cancer stem cell (CSC) markers. The CSC population is also thought to play a key role in breast cancer development, progression, and relapse following treatments [23-27]. Overexpression of EZH2 promotes self-renewal of breast tumor initiating cells [28]. Interestingly, in embryonic stem cells, EZH2 directly controls the expression of Oct4, a stem cell marker, to regulate the stem cell equilibrium [29]. Together, these evidences suggest that EZH2 may regulate cancer stem/initiating cell equilibrium in IBC.

In the present study, we examined the expression of the components of PRC2 in human IBC cells, as well as the effects of EZH2 knockdown on the formation of tumor spheroids, invasion and tumor growth of human IBC cells.

## Methods and materials

### Cell culture

SUM149 and SUM190 cells were cultured in Ham’s F-12 media supplemented with 10% fetal bovine serum (FBS), 1 μg/ml hydrocortisone, 5 μg/ml insulin and antibiotic-antimycotic. Primary human mammary epithelial cells (HMEC) were isolated and cultured as previously described [30]. The protocol was approved by institutional review board (IRB) of Fox Chase Cancer Center (FCCC).

The FC-IBC-02 tumor cells were derived from primary human breast cancer cells isolated from pleural effusion fluid of an IBC patient. Human samples used in this study were acquired with approval of the Fox Chase Cancer Center’s Institutional Review Board. Importantly, written informed consent form was obtained from each participant. FC-IBC-02 cells were cultured in Ham’s F12 with 10% FBS and 5 ml Insulin/L with 100 μg/L hydrocortisone and antibiotic-antimycotic. FC-IBC-02 cells grow as both adherent (FC-IBC-02A) and suspension (FC-IBC-02S) populations.

### EZH2 knockdown and lentivirus infection

The sense sequences of 2 individual 21-nucleotide shRNAs targeting the human EZH2 genes (shEZH2) were previously described [31]. Lentivirus packaging was performed using ViraPower system (Invitrogen) according to the manufacturer’s instruction as previously described [31]. Briefly, the cells were infected with lentivirus expressing shEZH2 or vector control. The infected cells were drug selected with 2 μg/mL of puromycin to eliminate non-infected cells.

### Antibodies and immunoblot

Immunoblot was performed using previously described methods [31,32]. In brief, cells were lysed in 1 × lysis buffer (Cell signaling) or 1 × SDS loading buffer. Tumor tissue was homogenized in 10 mM Tris–HCl (pH 7.8), the homogenate centrifuged at 10,000 rpm for 10 min at 4°C, and glycerol added (final concentration 15%) to the supernatant. Protein concentration was determined using the BCA protein assay reagent kit (Pierce, Rockford, IL). Equal amounts of protein from cell lysates or tumor tissue homogenates were resolved by SDS-PAGE electrophoresis. The membranes were incubated at 4°C overnight with the following antibodies: mouse anti-EZH2 (1:2,500; BD Bioscience), mouse anti-EED (1:2,000; Millipore), mouse anti-SUZ12 [31], mouse anti-β-actin (1:5,000; Santa Cruz). After incubation with anti-mouse IgG horseradish peroxidase conjugated secondary antibody (1:5,000; Amersham Pharmacia Biotech), immunoreactive proteins were visualized by the enhanced chemiluminescence reagents.

### Cell proliferation and tumor spheroid formation assays

Cells were infected with lentivirus encoding 2 individual shEZH2s or control for 48 hrs. Cell proliferation was monitored by absorbance using the MTS assay (CellTiter 96 AQueous One Solution cell proliferation assay, Promega) according to the manufacturer’s instruction. 2000 cells were seeded in triplicate in a 96-well plate. At the indicated times, absorbance at 490 nm was measured in a microplate reader.

For tumor spheroid formation, a total of 2000 single cell suspensions were plated into 24-well ultra-low attachment plates (Corning) and cultured in serum-free mammary epithelial growth medium (MEGM, BioWhittaker) supplemented with B27 (Invitrogen), 20 ng/mL epidermal growth factor (EGF), 40 ng/mL bFGF (BD Biosciences), and 4 μg/mL heparin (Sigma) [22]. After 6 days in culture, tumor spheroids were counted.

### Cell migration and invasion assays

For cell migration assay, 48 hrs after shRNA infection, SUM149 cells (7 × 10^4^) were suspended in 0.5 ml of Ham’s F-12 media and seeded onto the noncoated 8 μm membrane of cell culture insert (BD Bioscences) in a 24 well plate with Ham’s F-12 media supplemented with 10% FBS. For matrigel invasion, the membrane of cell culture insert was coated by adding 60 μl of 1:40 diluted matrigel basement membrane matrix (BD Bioscences) with Ham’s F-12 media. 1 × 10^5^ cells were suspended in 0.5 ml of Ham’s F-12 media and plated on top of the matrigel. After incubating 24 hrs, the cells were fixed with 4% formaldehyde and stained with 0.5% crystal violet in PBS. The number of cells that migrated across the membrane or invaded through the matrigel-coated membrane was determined in 9 fields across the center and the periphery of the membrane.

### In vivo tumorigenicity

The protocol was approved by FCCC institutional animal care and use committee (IACUC). Control or shEZH2 expressing SUM149 (3 × 10^6^, n = 6) and FC-IBC-02S (1 × 10^6^, n = 4) cells were suspended in 200 μL of 1:1 ratio of phosphate-buffered saline/matrigel (BD Biosciences) and orthotopically injected into the mammary fat pads of six week old female C.B-17 severe combined immunodeficient (SCID) mice. Tumor volume was calculated from the formula TV = L*W*H*0.5236 where L, W, and H are the tumor dimensions in three perpendicular dimensions by caliper measurement. Mice were sacrificed 6 weeks (SUM149) or 8 weeks (FC-IBC-02S) post inoculation. Tumors were surgically removed and weighed.

For *in vivo* angiogenesis assay, formalin-fixed paraffin-embedded tumors from shEZH2 or control groups were cut onto glass slides and processed for immunohistochemical (IHC) staining using previously described methods [31]. Purified rat anti-mouse CD31 antibody (BD PharMingen) was used for the staining. CD31-positive vessels were enumerated in randomly selected fields.

### Statistical analysis

Quantitative data were expressed as mean ± SEM. Analysis of variance (ANOVA) with Student’s t test was used to determine the significant differences among experimental groups, and *P* < 0.05 was considered significant.

## Results

### EZH2 is expressed at higher levels in human IBC cells

**Figure 1:**
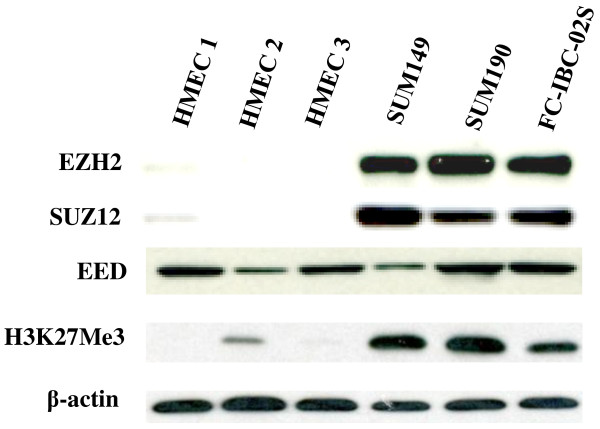
**EZH2 is expressed at higher levels in human IBC cell lines.** Expression of EZH2, SUZ12, EED, H3K27Me3, and β-actin in 3 indicated human IBC cell lines and 3 individual batches of primary normal human mammary epithelial cell cultures (HMEC) was examined by immunoblot analysis.

EZH2 was expressed frequently in IBC tumors and predicts worse clinical outcome of IBC patients [21]. We first sought to examine the expression of EZH2 and other essential components of PRC2 namely EED, and SUZ12 in 3 individual isolations of normal primary HME cells and 3 human IBC cell lines. The SUM149 and SUM190 cell lines are the only available human IBC cell lines that are widely used in IBC studies. FC-IBC-02 cell line was a newly derived IBC cell line using tumor cells from pleural effusion fluid of a patient with metastatic secondary IBC [22]. FC-IBC-02 cells were ER(-), Pgr(-) and HER2 non-amplified, and formed tumor spheroids in suspension culture that were strongly positive for E-cadherin, β-catenin adhesion molecules. The tumors were developed with lung micro metastasis when the cells were injected in the mammary fat pad of SCID mice and recapitulated the tumor emboli that is a signature of IBC in humans. Compared with normal HME cells, both EZH2 and SUZ12 were expressed at significantly higher levels in all three tested human IBC cell lines (Figure 1). Consistently, H3K27Me3, the product of EZH2 histone H3 lysine 27 methyltransferase activity, was also expressed at higher levels in IBC cell lines (Figure 1). Interestingly, there was no obvious difference in EED protein expression between normal HMECs and human IBC cell lines (Figure 1). Based on these results, we conclude that EZH2, SUZ12, and H3K27Me3 are expressed at higher levels in human IBC cells compared with normal HMEC.

### High tumorigenicity correlates with high expression of EZH2 in FC-IBC-02 cells

**Figure 2:**
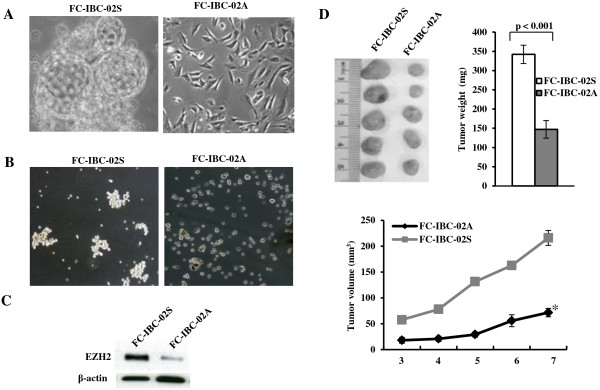
**High tumorigenicity correlates with high expression of EZH2 in FC-IBC-02 cells. A**. Initial FC-IBC-02 cells obtained from pleural effusion fluid of IBC patient growing as suspension (FC-IBC-02S) and adherent (FC-IBC-02A) populations (×200 magnification). Tumor spheroids exhibit budding from parent to daughter spheroid in suspension culture. **B**. Tumor spheroid growth of FC-IBC-02S and FC-IBC-02A cells in suspensions under ultra-low attachment culture condition for 6 days (×100 magnification). **C**. Expression of EZH2 in suspension and adherent populations of FC-IBC-02 cells was examined by immunoblot. **D**. Growth of FC-IBC-02 adherent (2 × 10^6^) and suspension (2 × 10^6^) cells *in vivo* in SCID mice. Tumor volume was monitored over time, and the tumor was excised and weighed after 7 weeks. *, *P* < 0.001 compared to FC-IBC-02A at week 7.

We observed that the newly derived FC-IBC-02 tumor cells grow as both adherent and suspension populations when they are initially obtained from pleural effusion fluid of the IBC patient. In addition, the suspension population of FC-IBC-02 cells spontaneously formed tumor spheroids, a characteristic of tumor stem/initiating cells (Figure 2A). When single cells were cultured in suspension under low adherence conditions, adherent cells formed less and smaller tumor spheroids compared to suspension cells (Figure 2B), suggesting that adherent cells are unable to self-renew. EZH2 has been implicated in regulating stem cell renewal, differentiation, and carcinogenesis [28,29]. Thus, we compared the expression of EZH2 in the initial adherent and suspension populations of FC-IBC-02 cells. Interestingly, the expression level of EZH2 was higher in suspension cell populations compared to adherent cell populations (Figure 2C). Next, we orthotopically transplanted the equal numbers (2 × 10^6^) of adherent and suspension FC-IBC-02 cells into the mammary fat pads of mice with 5 mice in each cell population. Tumor volume was monitored over time, and the tumor was excised and weighed after 7 weeks. Interestingly, the tumors were notably smaller from the transplanted adherent cell populations compared with suspension populations (Figure 2D). Together, our results suggest that expression levels of EZH2 positively correlates with tumor spheroid formation and tumorigenicity in this new IBC cell line.

### Knockdown of EZH2 inhibits the tumor spheroid formation of human IBC cells *in vitro* and tumor growth *in vivo*

**Figure 3:**
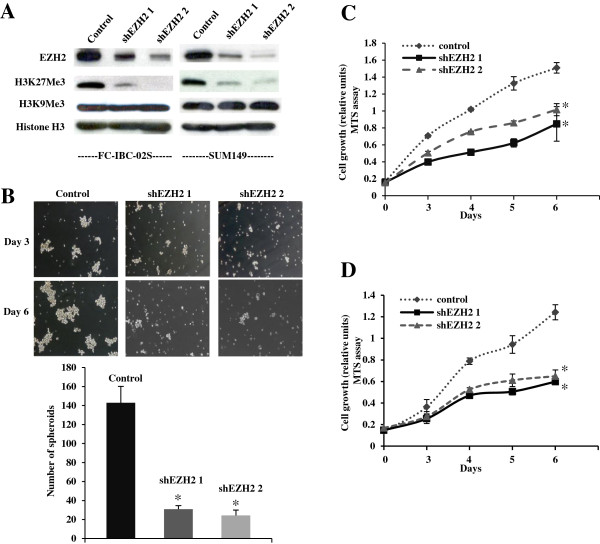
**EZH2 knockdown inhibits the cell growth of SUM149 and FC-IBC-02S cells and tumor spheroid formation of FC-IBC-02S cells. A**. SUM149 and FC-IBC-02S cells were infected with 2 individual lentivirus encoded shEZH2 or control. Expression of EZH2, H3K27Me3, H3K9Me3, and histone H3 was examined in drug-selected cells by western blotting. **B**. Same as **A**, 2000 of control or shEZH2 expressing FC-IBC-02S single cells were cultured in suspension in ultra-low attachment plates. After culturing 6 days, the number of tumor spheroids was counted. Mean of 3 independent experiments with SEM. *, *P <* 0.001 compared to control. **C** and **D**. Same as **A**, but 2 × 10^3^ of drug-selected cells were seeded and assayed by MTS in SUM149 **(C)** and FC-IBC-02S **(D)** cells at indicated time points. Mean of 3 independent experiments with SEM. *, *P <* 0.01 compared to control.

Since EZH2 was overexpressed in IBC cells and EZH2 expression positively correlates with tumor spheroid growth and tumorigenicity in FC-IBC-02 cells, we sought to determine the effects of EZH2 knockdown on tumor spheroid formation of IBC cells *in vitro* and the tumorigenicity of IBC *in vivo* in an orthotopic xenograft model in immunodeficient mice. To knockdown EZH2 expression in FC-IBC-02S and SUM149 cells, we used 2 individual shRNAs to the human *EZH2* gene (shEZH2). We first confirmed the knockdown efficacy of these shEZH2s in human IBC cell lines by immunoblotting (Figure 3A). Consistently, the level of H3K27Me3 expression was also significantly reduced by shEZH2 in both cell lines (Figure 3A). We compared the cell growth of control and shEZH2-infected IBC cells using the MTS assay. Compared with controls, EZH2 knockdown significantly suppressed the growth of human IBC cells (Figure 3C and D). We observed the growth inhibition by two individual shEZH2s, suggesting that the observed effects are likely not due to off-target effects. In addition, shEZH2 suppressed the growth of both SUM149 (Figure 3C) and FC-IBC-02S (Figure 3D) IBC cell lines, suggesting that the observed effects are not cell line specific. Importantly, we showed EZH2 knockdown significantly suppressed the tumor spheroid formation of FC-IBC-02S cells in suspension culture under ultra-low attachment culture condition, which is a characteristic of IBC cancer stem cells (Figure 3B). Based on these results, we conclude that knockdown of EZH2 suppresses the proliferation and tumor spheroid growth of human IBC cells *in vitro*.

**Figure 4:**
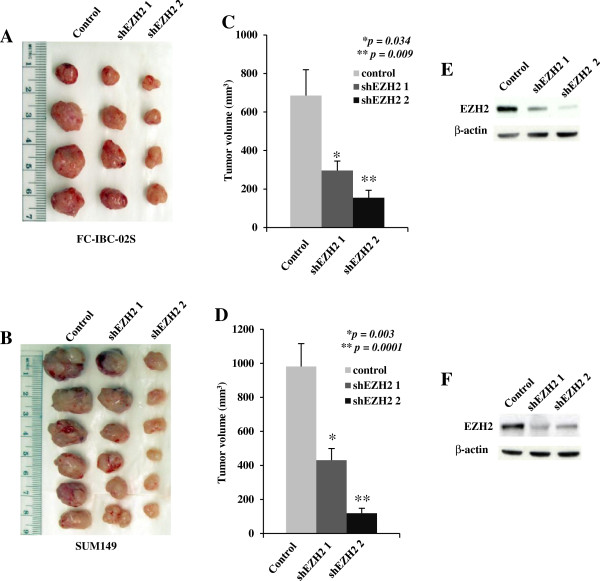
**EZH2 knockdown inhibits the growth of SUM149 and FC-IBC-02S cells *in vivo *in SCID mice. A** and **B**. Control or shEZH2 expressing FC-IBC-02S (1 × 10^6^) and SUM149 (3 × 10^6^) cells were orthotopically transplanted into the mammary fat pads of SCID mice. Nine weeks (FC-IBC-02S cells) and six weeks (SUM149 cells) post injection, tumors were removed from mice and imaged. **C** (FC-IBC-02S) and **D (**SUN149) cells, tumor size was measured by caliper measurements. Data shown represent mean tumor volume with SEM, *p* value compared with control. **E** (FC-IBC-02S) and **F** (SUN149), western blot assay of EZH2 expression from pooled FC-IBC-02S and SUM149 xenografted tumors.

We next sought to determine the effects of EZH2 knockdown on the tumorigenicity of human IBC cells *in vivo* in an orthotopic xenograft model in immunodeficient mice. Toward this goal, control and shEZH2-expressing FC-IBC-02S and SUM149 cells were orthotopically transplanted into the mammary fat pads of immunodeficient mice. Compared with controls, shEZH2 significantly suppressed the growth of xenografted human IBC cells in immunodeficient mice. Similar suppression of tumor growth by shEZH2 was observed for both xenografted FC-IBC-02S (Figure 4A and C) and SUM149 (Figure 4B and D) IBC cells, suggesting that the observed effects are not cell line specific. We confirmed the EZH2 knockdown in dissected tumors by immunoblotting (Figure 4E and F). Together, we conclude that EZH2 knockdown significantly inhibits the growth of human IBC cells *in vivo* in orthotopic xenograft IBC models.

### EZH2 knockdown inhibits the invasion and tumor angiogenesis of human IBC cells

**Figure 5:**
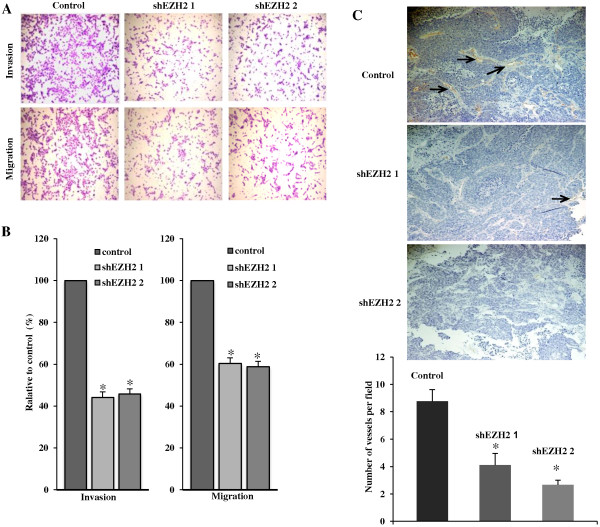
**EZH2 knockdown suppresses the invasion and tumor angiogenesis of human IBC cells. A**. Control and shEZH2 expressing SUM149 cells at equal number were seeded onto the noncoated membrane (migration) and matrigel-coated membrane (invasion). After incubating 24 hrs, the cells migrated through both uncoated and matrigel-coated membrane were stained with 0.05% crystal violet in PBS. **B**. Quantitation of **A**, relative percentage of shEZH2 expressing cells migrated through uncoated membrane or invaded through matrigel-coated membrane compared to controls was indicated. Mean of 3 independent experiments with SEM. *, *P <* 0.05 compared to control. **C**. Tumor tissues from FC-IBC-02S xenografts were stained with anti-CD31 antibody to measure microvessel density (×100 magnification). The CD31-positive microvessels are marked (arrows). Quantitation of CD31 vessel staining is shown in the bottom graph. *, *P* < 0.01 compared to control.

EZH2 has been implicated in regulating invasion of breast cancer cells [15]. Thus, we sought to determine the effects of EZH2 knockdown on the invasion of human IBC cells. Toward this goal, we tested the ability of human IBC cells infected with control or shEZH2-encoding lentivirus migrating through uncoated membrane or invading through matrigel-coated membrane using a Boyden-chamber invasion assay. Compared with controls, EZH2 knockdown significantly inhibited the migration and invasion of SUM149 cells (Figure 5A and B). We conclude that EZH2 knockdown inhibits the migration and invasion of human IBC cells. Interestingly, EZH2 knockdown tumors showed less blood vessels compared with control tumors (Figure 4A and B). To examine the reduced angiogenesis, we immunostained tumor sections from xenografted FC-IBC-02S with CD31, a marker used to quantitate vessel density. Compared with control tumors, EZH2 knockdown tumors displayed a significant reduction in CD31-positive vessels (Figure 5C). These results suggest that EZH2 knockdown inhibits tumor angiogenesis, and subsequently inhibits tumor growth.

## Discussion

The major finding of this study is that the EZH2 knockdown significantly suppressed the formation of tumor spheroids *in vitro* (a characteristic of IBC cancer stem cells), and tumor growth *in vivo* in a new human IBC model. New FC-IBC-02 cell line is the first to be used in studying the functional role of EZH2, although the negative effects of silencing of EZH2 on cell growth have been shown in some breast cancer cell lines including SUM149 cells.

We have recently shown that EZH2 is often expressed in IBC tumors and is associated not only with unfavorable prognostic variables but also with significantly worsening survival [21]. Consistent with previous findings, EZH2 was overexpressed in human IBC cell lines. These findings suggest that clinical outcomes of IBC patients can be stratified on the basis of EZH2 status. We further examined the function of EZH2 in SUM149 cells and a new IBC cell line-FC-IBC-02 derived from pleural effusion fluid of an IBC patient. A very recent study showed that SUM149 cell line might not be IBC-specific from gene expression data [33]. FC-IBC-02 cell line, however, is a more representative model for the IBC studies [22]. The treatment options for IBC patients, particularly for triple-negative IBC are very limited at present, while the results of this study indicate that EZH2 is a putative target for developing a new treatment for this devastating disease.

The mechanism of EZH2 promoting IBC remains to be elucidated. With IBC human cell lines in this study, we showed that both EZH2 and H3K27Me3 are expressed at higher levels compared to cultured normal HME cells (Figure 1). EZH2 catalyzes trimethylation of lysine 27 on histone 3, an epigenetic label mediating gene silencing, while the correlation between H3K27Me3 expression and clinical outcome is inconsistent. A very recent study showed that there was a significantly different expression of both EZH2 and H3K27Me3 in different subtypes of breast tumor [34]. High abundance of EZH2 was detected in basal-like, triple-negative and HER2-enriched tumors, but high H3K27Me3 was found mainly in luminal A, HER2-enriched and normal-like tumors. Further studies are warranted to determine the role of H3K27Me3 expression in IBC tumors.

Although there are few cell lines currently available to evaluate the biology of IBC, there is no good model available for CSC studies in IBC. The majority of IBC studies have been performed using the cell lines SUM149 and SUM 190, both of which were developed from the primary tumor of IBC patients [10]. Only the Mary-X and the WIBC-9 *in vivo* xenograft models are available to recapitulate the tumor emboli that are the signature of IBC in humans, but none of them can be successfully passaged or propagated long term *in vitro* cultures [35,36]. Our recent studies have shown that new developed FC-IBC-02 cells can be successfully passaged under both adherent and suspension culture conditions [22]. This new IBC model could be a more representative model for the further IBC studies since FC-IBC-02 cells were able to develop tumor with the presence of tumor emboli and metastasis in SCID mice. In this study, we are interested in studying the functional role of EZH2 in this new IBC model. We observed that newly derived FC-IBC-02 suspension cell population formed tumor spheroids, a characteristic of CSC and expressed a higher level of EZH2 and generated larger tumor in size compared to initial adherent cell population when transplanted into the mammary fat pads of SCID mice (Figure 2D). Notably, knockdown of EZH2 significantly inhibits the tumor spheroid growth of human IBC cells *in vitro.* Recent studies showed that CSC population may be responsible for resistance to chemotherapy, radiation therapy and contribute to relapse following the treatment [25-27,37,38]. This suggests that more effective therapies will require the successful targeting of the CSCs population. We believe that the FC-IBC-02 cell line described in this study could serve as a potential model for CSC studies in human IBC. The findings of this study provide evidence that EZH2 is a potential target for the self-renewal of IBC cells.

Angiogenesis is required for invasive tumor growth and metastasis. A recent study showed that EZH2 is a key regulator of tumor angiogenesis through its expression in cancer associated endothelial cells in human ovarian cancer [39]. Interestingly, we observed a visible evidence for the loss of angiogenesis in the EZH2 knockdown tumors (Figure 4A and B). In addition, there was a significant reduction in CD31-positive vessels in EZH2 knockdown tumors compared to control tumors (Figure 5C), suggesting that the loss of angiogenesis subsequently inhibits tumor growth. The mechanisms implicated in the angiogenesis role of EZH2 in IBC need further investigation. Several specific inhibitors of EZH2’s methyltransferase activity have been reported [40-42]. Our recent studies have shown that the three-dimensional culture sensitizes epithelial ovarian cancer cells to EZH2 methyltransferase inhibition, which suppresses cell growth, induces apoptosis and inhibits invasion [43]. Further studies are warranted to examine the effects of these specific inhibitors of EZH2 on the characteristics of IBC, such as homotypic aggregation of tumor emboli, invasion and metastasis of IBCs.

## Conclusions

EZH2 are overexpressed in human IBC cell lines. EZH2 is critical for the formation of tumor spheroids and invasion of human IBC cells and could be a potential target for developing novel therapeutic strategies for human IBC.

## Abbreviations

IBC: Inflammatory breast cancer; EZH2: Enhancer of zeste homolog 2; shEZH2: short hairpin RNA targeting the human EZH2 genes; CSC: Cancer stem cell.

## Competing interests

All other authors declare no competing interests.

## Authors’ contributions

ZM and HL performed all the experiments, analyzed the data and drafted the manuscript. SF assisted with in vivo experiments. KA assisted with establishing IBC cell line. MC and RZ conceived of the study, reviewed and finalized the manuscript. All authors read and approved the final manuscript.
